# Natural Variation in Physiological Responses of Tunisian *Hedysarum carnosum* Under Iron Deficiency

**DOI:** 10.3389/fpls.2018.01383

**Published:** 2018-10-02

**Authors:** Heithem Ben Abdallah, Hans Jörg Mai, Tarek Slatni, Claudia Fink-Straube, Chedly Abdelly, Petra Bauer

**Affiliations:** ^1^Institute of Botany, Heinrich Heine University Düsseldorf, Düsseldorf, Germany; ^2^Laboratory of Extremophile Plant, Center of Biotechnology of Borj Cedria, Hammam-Lif, Tunisia; ^3^INM-Leibniz Institute for New Materials, Saarbrücken, Germany; ^4^Cluster of Excellence on Plant Sciences, Heinrich Heine University, Düsseldorf, Germany

**Keywords:** legume, natural diversity, iron deficiency, chlorophyll, acidification, Fe reductase activity, *IRT1*

## Abstract

Iron (Fe) is an essential element for plant growth and development. The cultivation of leguminous plants has generated strong interest because of their growth even on poor soils. Calcareous and saline soils with poor mineral availability are wide-spread in Tunisia. In an attempt to select better forage crops adapted to Tunisian soils, we characterized Fe deficiency responses of three different isolates of *Hedysarum carnosum*, an endemic Tunisian extremophile species growing in native stands in salt and calcareous soil conditions. *H. carnosum* is a non-model crop. The three isolates, named according to their habitats Karkar, Thelja, and Douiret, differed in the expression of Fe deficiency symptoms like morphology, leaf chlorosis with compromised leaf chlorophyll content and photosynthetic capacity and leaf metal contents. Across these parameters Thelja was found to be tolerant, while Karkar and Douiret were susceptible to Fe deficiency stress. The three physiological and molecular indicators of the iron deficiency response in roots, Fe reductase activity, growth medium acidification and induction of the *IRON-REGULATED TRANSPORTER1* homolog, indicated that all lines responded to -Fe, however, varied in the strength of the different responses. We conclude that the individual lines have distinct adaptation capacities to react to iron deficiency, presumably involving mechanisms of whole-plant iron homeostasis and internal metal distribution. The Fe deficiency tolerance of Thelja might be linked with adaptation to its natural habitat on calcareous soil.

## Introduction

Iron (Fe) is an essential micronutrient with numerous cellular functions, e.g., in photosynthesis, respiration, DNA synthesis, and N_2_ fixation. Plants are frequently challenged by Fe deficiency, especially on alkaline and calcareous soils due to poor Fe solubility under these conditions. In Tunisia, the exploration of such kinds of natural habitats and saline environments revealed that they are colonized by a native leguminous vegetation which might have specific adaptations to both, salinity and nutrient deficiencies, especially Fe (Ben Abdallah et al., 2017). Leguminous plants take up reduced Fe using mainly the so-called Strategy I. The main feature of Strategy I plants, e.g., in *Arabidopsis thaliana* and leguminous plants, is that they acidify the soil via proton extrusion through an ATPase, reduce ferric to ferrous Fe by a ferric chelate reductase and take up the divalent Fe via divalent metal IRON-REGULATED TRANSPORTER1 (Brumbarova et al., 2015), being a member of the ancient ZIP ( = ZRT/IRT1) protein family (Eng et al., 1998). *IRT1* homologs were found Fe-regulated in roots of multiple legumes like *Pisum sativum* (Cohen et al., 2004), *Medicago truncatula* (Lopez-Millan et al., 2004), *Arachis hypogaea* (Ding et al., 2010), *Glycine max* (Brear et al., 2013), and *Vigna radiata* (Muneer et al., 2014).

Natural variation studies make use of the existing natural allelic diversity in plant populations as a source to pinpoint the adaptive alleles for relevant traits. Natural variation was successfully applied in model plants to identify causal alleles by genome-wide association studies for such different traits as environmental adaptation in Arabidopsis (Li et al., 2010), or nutritional quality and agronomic traits in maize (Diepenbrock et al., 2017) and rice (Si et al., 2016). The model legumes *M. truncatula* and *G. max* are particularly suited for natural biodiversity studies (Gentzbittel et al., 2015). Prerequisites for association studies are genome sequence variation reflected by a broad collection of ecotypes demonstrating phenotypic diversity for given traits. However, alternative procedures are available for studying natural diversity of small population collections in the absence of large genome sequence data, e.g., by making use of recombinant or near-isogenic inbred lines suitable for mapping and gene identification (Yan et al., 2017), by isolating candidate genomic regions and genes based on comparative genomics (Friesen et al., 2010, 2014; Turner et al., 2010) and transcriptomics or proteomics of genetically divergent lines (Voelckel et al., 2017).

In Tunisia, a large area is arid to semi-arid with calcareous and alkaline-saline soil conditions, where bioavailability of Fe, Mg, and other minerals is low, posing a major problem for crop yield (Rabhi et al., 2007). Such areas are often used for forage crop and cattle production. To improve agricultural land usage and provide better perspectives to farmers, there is a need to select tolerant crops adapted to such poor soils.

Perennial *Hedysarum carnosum* was proposed as a prospective well-palatable pasture crop being a naturally adapted halophyte to the Mediterranean basin with good potential for rehabilitation strategies (Le Houerou, 1996). The Hedysarum (sweetvetch) genus belongs to the Fabaceae plant family, which contains many of the very important crops. Species of this genus have arisen in a non-monophyletic manner, as recently established based on multiple sequence alignments and phylogenetic tree constructions of 58 accessions accounted to this genus using nuclear and plastid gene sequences (Liu et al., 2017). *H. carnosum* (also known as *Sulla carnosa*) that is subject of this study is most related to *Hedysarum coronarium* (also known as *Sulla coronaria*) (Liu et al., 2017). *H. coronarium* is wide-spread around the Mediterranean basin. In contrast, *H. carnosum* is endemic in Tunisia where it grows in different climates, ranging from semi-arid (Karkar) to arid regions (Thelja and Douiret). This species prefers slightly acid to alkaline soils (pH 5.5–8.5), sandy loams and clays, and good growth is achieved on alkaline soils. As an extremophile, *H. carnosum* also grows on Tunisian saline sodic soils, represented especially by Chotts and Sebkhas (Dallali et al., 2012). This species plays important roles in animal feed due to its high protein contents and tannins (Aissa et al., 2016). *H. carnosum* is a non-model crop and molecular investigation is difficult because of the lack of molecular data and gene sequences. *H. carnosum* responds to salt, potassium and magnesium deficiencies and low Fe availability (Farhat et al., 2016; Elkhouni et al., 2017; Hafsi et al., 2017). However, in all these studies only a single *H. carnosum* line was studied, rendering it difficult to judge the natural adaptation potential within the species. Moreover, these studies primarily focused on photosynthetic parameters in leaves. The objective of this work was to investigate the effect of Fe deficiency at the physiological level in *H. carnosum* and to compare the responses in three different isolates collected from different natural sites. Our data show that the Thelja isolate is the most tolerant to Fe deficiency stress and therefore will be the best choice for use in future genetic and RNAseq studies to identify the natural basis for calcareous soil-induced Fe deficiency. Thelja will also represent the most promising ecotype for agricultural purposes.

## Materials and Methods

### Plant Material and Growth Condition

Three isolates of *H. carnosum* were acquired by collecting seeds from Karkar, Thelja, and Douiret in Tunisia (see **Supplementary Figure 1** for species characteristics and geographic repartition of collected isolates). About 300 seeds per isolate were collected from an average of 10 plants distributed in a diameter of 100 m. Seeds of the three isolates were germinated and grown in separate green houses. Due to allogamy plants were multiplied by cross-fertilization of plants within each isolate. The F3 generation was used for analyses.

Seeds were mechanically scarified by rubbing in between fine grit sand paper sheets. The seeds were sterilized in 10% sodium hypochlorite for 8 min and then abundantly rinsed with distilled water. After a 10 min imbibition phase, they were germinated for 4 days at 20°C in Petri dishes on constantly moistened filter paper.

Four day-old seedlings were transferred to a half strength aerated liquid nutrient solution for 2 days. Similarly sized seedlings were then selected and cultured in groups of 4 or 10 plants in 1 or 10 L of full strength aerated nutrient solution [1.5 mM Ca(NO_3_)_2_, 1.25 mM KNO_3_, 0.75 mM MgSO_4_, 0.5 mM KH_2_PO_4_ and 10 μM H_3_BO_3_, 1 μM MnSO_4_, 0.5 μM ZnSO_4_, 3 μM M_O_O_4_Na_2_, 0.5 μM CuSO_4_, and 50 μM Fe-EDTA]. At day 6, the following treatments were conducted for the amount of time indicated in the text and figure legends: +Fe, control (Fe-sufficient medium with 50 μM Fe) and -Fe, Fe deficiency (medium without Fe). The pH was adjusted to 6.0 with NaOH for both, the +Fe (control) and -Fe (iron deficiency) treatments. Aerated hydroponic cultures were maintained in a growth chamber with a day/night regime of 16/8 h light-dark-cycle, a 24/18°C temperature cycle and a constant relative humidity of 70%. The solution was renewed every 4 days. The standard experiment was conducted using ten-day Fe sufficiency and deficiency treatments.

### Morphological Root and Shoot Phenotypes

**FIGURE 1:**
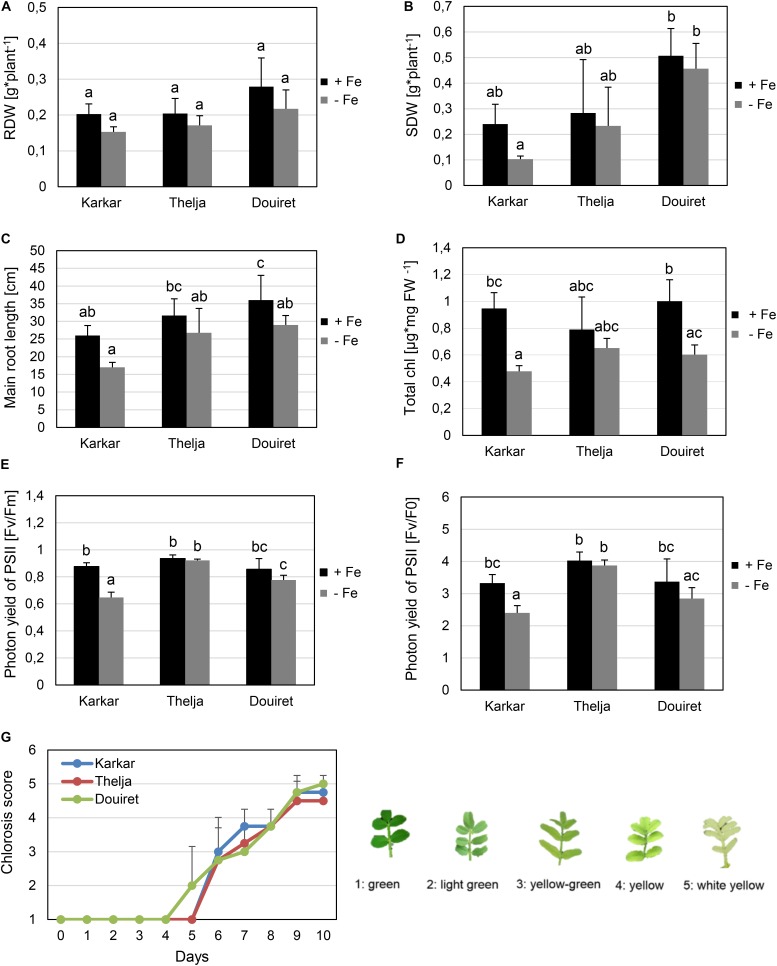
Effect of Fe deficiency stress on Karkar, Thelja, and Douiret lines. **(A)** Root dry weight (RDW), *n* = 3; **(B)** shoot dry weight (SDW), *n* = 3; **(C)** main root length, *n* = 4–5; **(D)** total chlorophyll (Chl) content, *n* = 3; **(E,F)** photon yield of PSII calculated by Fv/Fm and Fv/F_0_, *n* = 4; **(G)** chlorosis phenotype symptoms of young leaves calculated according to the indicated scale from 1, green leaves up to 5,white-yellow leaves, *n* = 4. *H. carnosum* plants were exposed for 10 days **(A–F)** or up to 10 days **(G)** to Fe-sufficient (+Fe) and Fe-deficient (–Fe) hydroponic growth conditions. Data are means ± SD; means with the same letter are not significantly different with *P* ≤ 0.05 according to ANOVA and Tukey’s HSD test.

Roots and shoots were harvested, dried in an oven at 70°C for 48 h and the dry weights determined per plant. The main root lengths were measured. The degree of leaf chlorosis was assessed in the youngest expanded leaves. The leaf chlorosis scale was determined as previously described (Schuler et al., 2012), ranging from 1 = green, 2 = light green, partially yellow, 3 = yellow-green, 4 = yellow to 5 = white-yellow, as shown in **Figure 1G**.

### Chlorophyll Measurements

Total chlorophyll was extracted from fresh leaves in 80% acetone and assayed photometrically at 645 nm and 663 nm. The OD values were used to calculate the total chlorophyll content in mg/g fresh weight of the leaves as published (Arnon, 1949).

### Pulse Amplitude Measurements (PAM)

Pulse Amplitude Measurements (PAM) was determined with the FluorCam FC 800-C machine (Photon Systems Instruments^TM^). Plants were adapted to darkness for about 15 min. Then single leaves were measured for F_0_ (minimal fluorescence) up to Fm (maximal fluorescence). To analyze photosystem II activity, Fv/Fm values and Fv/F_0_ values were calculated (Murchie and Lawson, 2013). F_0_ and Fm represent the minimum and maximum values of chlorophyll fluorescence, while F_V_ is the variable fluorescence.

### Acidification of the Growth Medium

The acidification capacity was determined after placing plants into 1 L nutrient solution (at pH 6.2), respectively, and measuring the pH of the nutrient solution in the subsequent days as indicated in the text and figure legend.

### Measurements of Root Fe Reductase Activity

Intact root systems were washed with 100 mM Ca(NO_3_)_2_ solution and submerged in the Fe reductase assay solution containing 0.1 mM Fe^3+^-NaEDTA and 0.3 mM ferrozine at pH 5.0 for 1 h in the dark. Then the absorbance was determined at 562 nm. The concentration of the Fe^2+^-ferrozine complex was calculated using the molar extinction coefficient of 28.6 mM^-1^ cm^-1^. The amount of Fe^2+^ was normalized to the root weight in the assay and Fe reductase activity was calculated.

### Mineral Element Analysis

To determine the metal ion content the youngest expanded leaves of *H. carnosum* were harvested and dried over 72 h at 70°C. After drying, the harvested leaves were finely powdered with an achat mortar and pestle. Metal contents (Zn, Fe, Cu) were determined using inductively-coupled plasma optical emission spectrometry (ICP-OES) at the Leibniz Institute for New Materials (INM, Saarbrücken).

### Obtention of *H. carnosum* cDNA Sequences and Multiple Sequence Alignment of Amino Acid Sequences

Obtention of *H. carnosum* cDNA sequences is outlined in **Supplementary Figure 2A**. *A. thaliana*, *M. truncatula*, *G. max*, *Lotus japonicus* sequences of *IRT1* and β-*ACTIN* (*ACT*) were aligned. Conserved regions near the start and stop codons were identified and primers matching 100% the *M. truncatula* sequences were designed (Mt primers, **Supplementary Figure 3**). With these Mt primers 1 μL of template root cDNA of *H. carnosum* was used to amplify the *IRT1* and *ACT* internal coding sequences in a standard PCR. PCR amplicon bands were purified from agarose gels according to standard procedures and sequenced.

Next, TAIL-PCR (**Supplementary Figure 2B**) was used to identify the unknown upstream 5^′^ and downstream 3^′^ cDNA sequences adjacent to the determined *HcIRT1* and *HcACT* partial sequences. We used three nested specific primers (S1–S3) that aligned near the edge of the known cDNA (**Supplementary Figure 3**). For extension in the opposite direction AD (arbitrary degenerate) primers were used. The AD primers were 64x–256x degenerate and designed to be relatively short (15–16 nt) with a low melting temperature (ca. 40–50°C) (**Supplementary Figure 3**). Three consecutive TAIL-PCR reactions were conducted as described (Liu et al., 1995). The third step PCR products were sequenced and *HcIRT1* and *HcACT* sequences assembled and provided to GenBank (accession numbers MH879027 and MH879028).

Multiple sequence alignment and construction of neighbor-joining trees using amino acid sequences was performed using the Clustal Omega tool at https://www.ebi.ac.uk.

### RNA Isolation and Quantitative Real-Time PCR

Gene expression was analyzed using three biological replicates. RNA isolation and reverse transcription-quantitative PCR were carried out as described previously (Ben Abdallah and Bauer, 2016). Briefly, total RNA prepared from 100 mg *H. carnosum* root tissue was used for cDNA synthesis using an oligo-dT primer. qPCR was conducted using the SYBR Green detection method. RT-qPCR primers were used for qPCR. The absolute quantity of initial transcripts was determined for the genes *IRT1* and *ACT* by standard curve analysis using mass standards prepared from *H. carnosum* cDNA PCR products amplified with Mt primers. Absolute expression data of *IRT1* was obtained after normalization to the internal control *ACT* gene. Each biological cDNA sample was tested in two technical qPCR replicates.

### Statistical Analysis

Morphological, physiological, and molecular data were obtained in at least three biological replicates, as detailed in the figure legends. Data of biological replicates were used to calculate mean values and standard deviations. Statistical significance was determined by applying *t*-tests (for two sample comparisons) and One-way ANOVA followed by Tukey’s HSD test (for more than two sample comparisons) designated as “ANOVA and Tukey’s HSD test” in the figure legends.

## Results

### Morphological and Physiological Shoot Responses to Fe Deficiency

Seeds from *H. carnosum* plants were collected in three different locations in Tunisia characterized by semi-arid, arid and Saharan conditions, named Karkar, Thelja, and Douiret, with saline-sodic, calcareous and sandy soil characteristics (**Supplementary Figure 1**). After seeds were multiplied for three generations, morphological and physiological experiments were carried out. We were interested in obtaining an ecotype with high tolerance to prolonged Fe deficiency growth conditions, a trait expected to be beneficial upon growth on calcareous soil. We therefore hypothesized that the three isolates might show different adaptation and respond differently to Fe deficiency conditions. Plant seedlings were grown in controlled hydroponic conditions and exposed to sufficient iron (+Fe) or deficient iron supply (-Fe) for 10 days. At first, we compared the tolerance/sensitivity of the lines to -Fe by measuring different growth parameters. The three isolates did not behave any different from each other in terms of root biomass production under + and -Fe (**Figure 1A**). Also in terms of shoot biomass production, the ecotypes were very similar (**Figure 1B**). Only one comparison resulted in a significant difference in biomass, which was the fourfold higher shoot dry weight of Douiret versus Karkar at -Fe (**Figure 1B**). However, none of the lines showed lower root or shoot biomass when grown at – compared to +Fe (**Figures 1A,B**). Karkar displayed a shorter main root compared to Douiret at +Fe. When comparing the main root length at -Fe versus +Fe, there was a significant decrease only in the case of Douiret but not Karkar and Thelja (**Figure 1C**).

Fe is required in high amounts during plant growth in the leaves to sustain photosynthesis and for chlorophyll synthesis. Fe can also be stored in chloroplasts in the form of ferritin. Lack of Fe results in the typical leaf chlorosis symptoms especially in the expanding leaves. Leaf chlorosis is caused by low chlorophyll contents under Fe deficiency. Karkar and Douiret had higher total chlorophyll contents at + than at -Fe (**Figure 1D**). Thelja, on the other hand, displayed no significant difference at + versus -Fe (**Figure 1D**). No significant differences were detectable between the lines at either + or -Fe (**Figure 1D**). PAM measurements based on chlorophyll fluorescence are an indicator for the photosynthetic performance under stress conditions. Low Fv/Fm and Fv/F_0_ ratios are indicative of stress affecting negatively the photosystem activity. We found that Karkar had lower Fv/Fm and Fv/F_0_ ratios at – versus +Fe, while no significant differences were found in Thelja and Douiret (**Figures 1E,F**). When comparing the lines with each other, Karkar had a lower Fv/Fm ratio than Thelja and Douiret and Thelja had a higher Fv/F_0_ ratio than Karkar and Douiret (**Figures 1E,F**). We were also interested in comparing the development of the leaf chlorosis during the 10 days of exposure to -Fe. Leaf chlorosis started 2 days earlier in Douiret than in Karkar and Thelja, but after 10 days the chlorosis had reached similar levels, as determined above from the chlorophyll measurements (**Figures 1D,G**).

**FIGURE 2:**
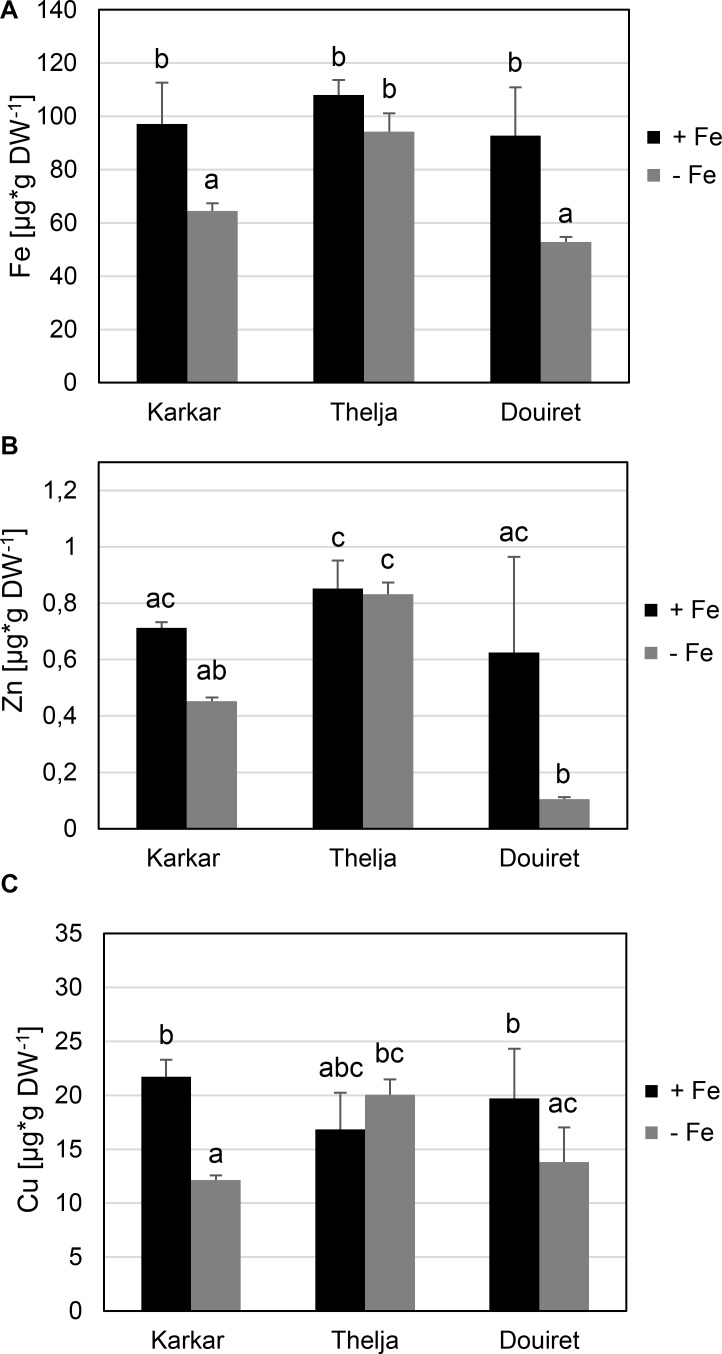
Metal contents of Karkar, Thelja, and Douiret. **(A)** Fe; **(B)** Zn; **(C)** Cu contents of the youngest expanded leaves. *H. carnosum* plants were exposed for 10 days to Fe-sufficient (+Fe) and Fe-deficient (–Fe) hydroponic growth conditions. Data are means ± SD; *n* = 4; means with the same letter are not significantly different with *P* ≤ 0.05 according to ANOVA and Tukey’s HSD test.

Next, we investigated whether the observed leaf chlorosis and impact on photosynthesis could be related to the amount of Fe taken up. We determined metal contents in the expanding leaves, as these are the organs where Fe deficiency symptoms are noted. Roots were not used since under hydroponic growth Fe is available in the Form of Fe Na EDTA, resulting in an accumulation and high Fe content, e.g., in the apoplasts of roots. In addition to Fe we measured Zn and Cu contents. Arabidopsis IRT1 can take up Zn but not Cu (Vert et al., 2002) and MtZIP6 can also transport Zn (Lopez-Millan et al., 2004). Karkar and Douiret had lower Fe contents upon -Fe than under +Fe, but not Thelja, which had comparable levels under both conditions (**Figure 2A**). Thelja also had higher Fe contents upon -Fe compared to Karkar and Douiret (**Figure 2A**). The Zn content was decreased at – versus +Fe growth conditions only in Douiret (**Figure 2B**). However, Thelja had a higher Zn content than Karkar and Douiret at – but not +Fe (**Figure 2B**). The Cu content was decreased at – versus +Fe in Karkar and Douiret, but again not in Thelja (**Figure 2C**). When comparing the lines with each other, a significant difference of Cu was only found in the comparison of Thelja versus Karkar at -Fe (**Figure 2C**).

**FIGURE 3:**
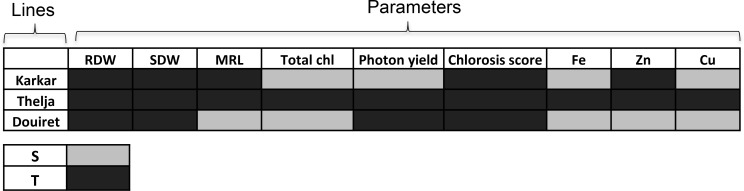
Summary assessment of Karkar, Thelja, and Douiret for Fe deficiency stress responses and metal contents. The investigated parameters are root and shoot dry weights (RDW, SDW), total chlorophyll (Chl), photon yield, chlorosis score (see **Figure 1**) and Fe, Zn, and Cu contents (see **Figure 2**). Responses that showed a decrease at – versus +Fe are designated by light gray color (S, sensitive) and no decrease by dark gray color (T, tolerant).

To conclude from this physiological and growth analysis of the three lines exposed to + and -Fe, we summarized the comparative outcomes for the parameters measured at + versus -Fe for each line and designated a significant decrease at – versus +Fe as “sensitive” and no decrease as “tolerant” behavior (**Figure 3**). Karkar received four sensitivity and five tolerance labels, Douiret six sensitivity and three tolerance labels, and Thelja nine tolerance labels. Root and shoot biomass were not identified as parameters that could be used to discriminate the behavior of the lines at + and -Fe, while leaf chlorosis, photosystem activity and metal contents were well suited to do so. Taken together, it can be deduced that Thelja shows tolerance to -Fe in contrast to the other two lines.

### Physiological and Molecular Root Responses to Fe Deficiency

**FIGURE 4:**
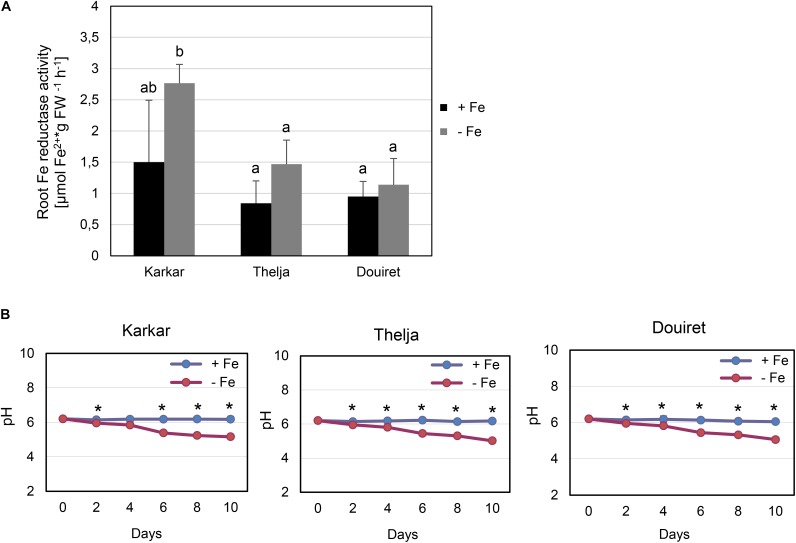
Physiological Fe deficiency responses of Karkar, Thelja, and Douiret. **(A)** Root Fe reductase activity, *n* = 4–5; **(B)** pH changes in the growth medium, *n* = 4. *H. carnosum* plants were exposed for 10 days **(A)** or up to 10 days **(B)** to Fe-sufficient (+Fe) and Fe-deficient (–Fe) hydroponic growth conditions. Data are means ± SD; means with the same letter are not significantly different with *P* ≤ 0.05 according to ANOVA and Tukey’s HSD test, means with ^∗^ label in B show a significant difference of + versus –Fe with *P* ≤ 0.05 according to a *t*-test.

**FIGURE 5:**
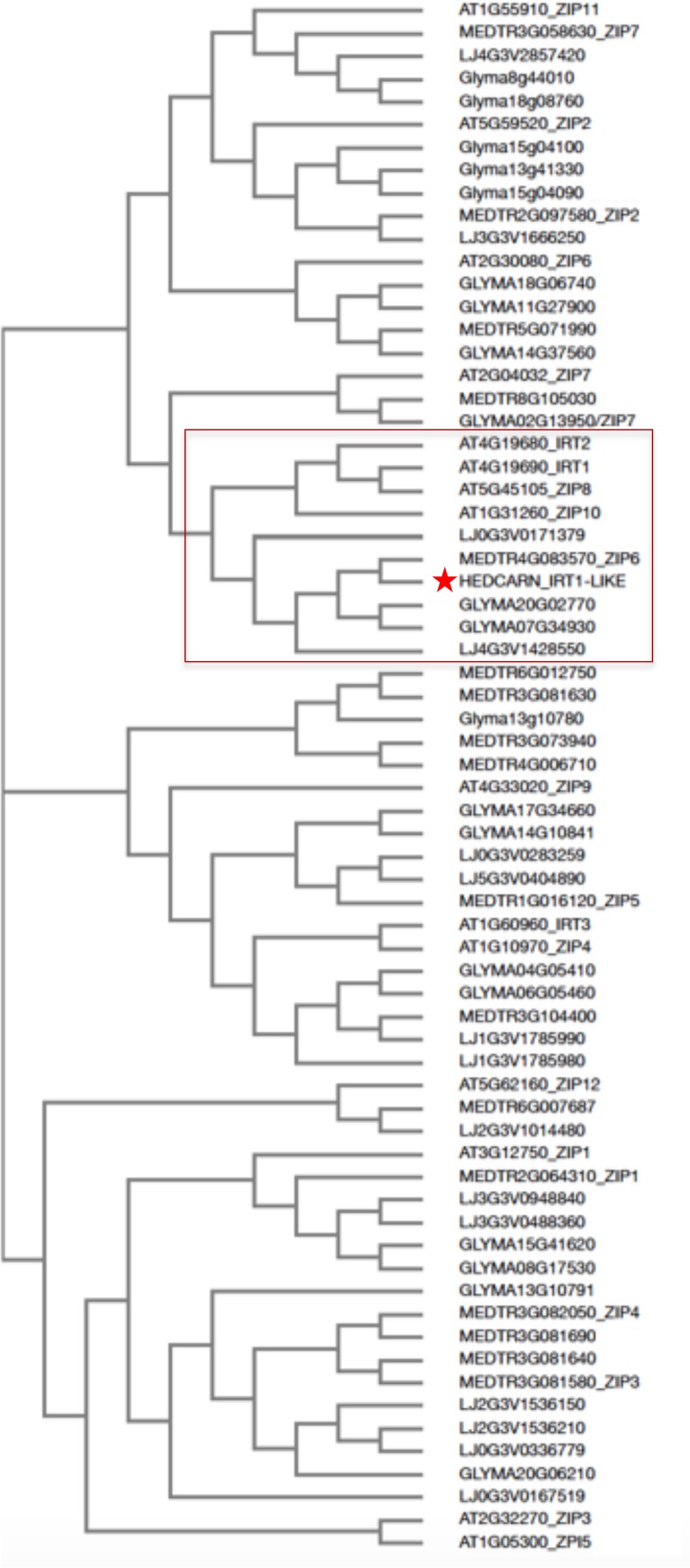
Neighbor-joining tree of HcIRT1 and ZIP protein sequences from selected legumes and Arabidopsis. The multiple amino acid sequence alignment was produced with *H. carnosum* IRT1 and all identified ZIP protein sequences from *A. thaliana*, *M. truncatula*, *G. max*, and *L. japonicus*. The box indicates the closest relatives of AtIRT1. The star indicates HcIRT1.

**FIGURE 6:**
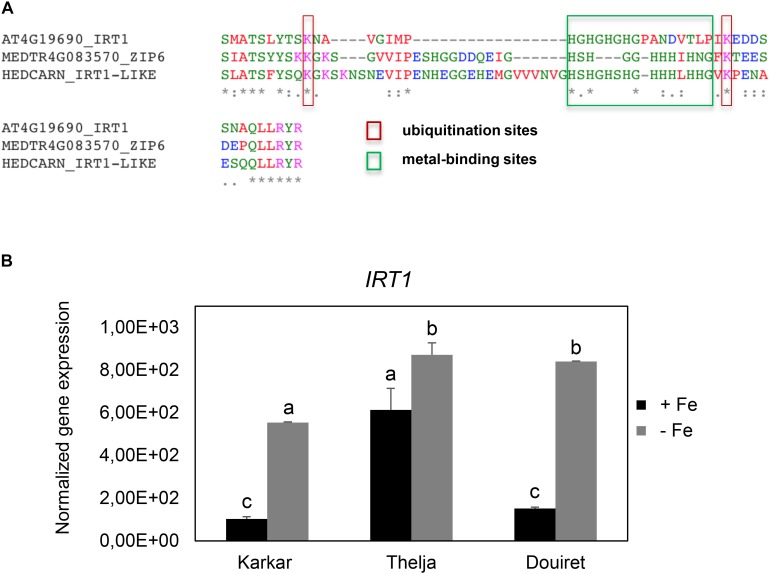
Conservation of functional IRT1 sequences and *IRT1* gene expression in Karkar, Thelja, and Douiret. **(A)** Multiple amino acid sequence alignment of the variable cytoplasmic loop region of AtIRT1, HcIRT1, and MtZIP6, containing relevant functional residues involved in ubiquitination (lysines) and metal binding (histidine stretch). **(B)** Normalized gene expression of *IRT1*. *H. carnosum* plants were exposed for 10 days to Fe-sufficient (+Fe) and Fe-deficient (–Fe) hydroponic growth conditions. Data are means ± SD; *n* = 3; means with the same letter are not significantly different with *P* ≤ 0.05 according to ANOVA and Tukey’s HSD test.

Roots of strategy I plants show typical Fe deficiency symptoms like enhanced soil acidification, Fe reduction and increased *IRT1* gene expression. Quantification of these responses is used to judge the degree of tolerance to Fe deficiency (Brumbarova et al., 2015). Therefore, we tested next for potential differences in the level of Fe deficiency responses in the root. None of the plants subjected to Fe deficiency showed a significant increase in root Fe reductase activity (**Figure 4A**). In a time-course experiment we found that the growth medium was acidified significantly starting as early 2 days after exchange to Fe deficiency and continued until 10 days in all three lines (**Figure 4B**). *H. carnosum* gene sequences were not deposited in the database. To conduct gene expression analysis by the RT-qPCR method, we selected *IRT1* as a target gene to reflect molecular Fe uptake regulation. Gene expression of additional genes were not investigated here as it is more useful to conduct RNAseq studies in the future. First, we identified homologs of *IRT1* and of the reference gene β-*ACTIN* (*ACT*) from *H. carnosum* using PCR and TAIL-PCR by exploiting sequence similarities among leguminous plant IRT1 sequences and available microarray-based gene expression data for *M. truncatula* (see section “Materials and Methods”; outline in **Supplementary Figure 2**). Since we found no differences in the amino acid sequences of IRT1 between the three *H. carnosum* lines, we compared the sequence to ZIP sequences from Arabidopsis and other legumes. The full-length HcIRT1 amino acid sequence was found most related to MtZIP6 in a neighbor-joining tree derived from a multiple sequence alignment of the entire families of *A. thaliana*, *M. truncatula*, *G. max,* and *L. japonicus* ZIP protein sequences (**Figure 5**). MtZIP6 was the only *M. truncatula* ZIP protein with high sequence similarity to HcIRT1 and AtIRT1 (**Figure 5**). MtZIP6 is up-regulated by -Fe in roots (He et al., 2009; Benedito et al., 2010) and it was characterized as Fe transporter (Lopez-Millan et al., 2004). All other *M. truncatula* ZIP proteins group along with other branches of *A. thaliana* ZIP proteins (**Figure 5**), indicating that these other MtZIP proteins have different functions in metal homeostasis. Interestingly, this analysis also shows that *A. thaliana* has a high expansion of four IRT1-like proteins (IRT1, IRT2, ZIP8, ZIP10). In this same branch of IRT1-like sequences, there are two *G. max*, two *L. japonicus* and only one *M. truncatula* ZIP sequence (highlighted by a red box in **Figure 5**). Thus, legumes have fewer IRT1-like proteins than Arabidopsis. One possible explanation could be the different genome duplication histories during evolution. HcIRT1 and MtZIP6 share two important functional sequence features in the predicted variable cytoplasmic loop region with AtIRT1, namely two conserved lysine positions used for ubiquitination in metal-directed IRT1 turnover (Kerkeb et al., 2008) and the histidine-rich stretch for metal-binding relevant for metal import by ZIPs into the cell (Zhang et al., 2017; **Figure 6A**). Hence, the sequence analysis convincingly suggests that *HcIRT1* encodes a functional IRT1 homolog. *HcIRT1* gene expression was found significantly induced by -Fe in Karkar, Thelja, and Douiret (**Figure 6B**). Thelja displayed a higher base level of *HcIRT1* expression in the +Fe control situation compared to Karkar and Douiret (**Figure 6B**). Thelja and Douiret had a higher *HcIRT1* expression level at -Fe versus Karkar (**Figure 6B**).

In summary, the three lines displayed root Fe deficiency response reactions which were most pronounced in case of *HcIRT1* induction and medium acidification, while Fe reductase activity increases were not found to be significant. Perhaps the constitutively elevated *IRT1* expression level of Thelja is linked with its higher Fe content under -Fe as an adaptation to growth on calcareous soil.

## Discussion

Here, we show that the extremophile *H. carnosum* shows natural variation and phenotypic plasticity with regard to Fe deficiency responses in 8 out of 12 measured parameters. This species is an endemic growing on Tunisian saline and calcareous soil conditions that are known to affect micronutrient use efficiency. Overall, Thelja is the most tolerant isolate showing tolerance to Fe deficiency perhaps as a consequence of its adaptation to calcareous soils.

All *H. carnosum* isolates sensed Fe deficiency and responded to this stress, while the outcome of -Fe stress was different among the ecotypes. The common -Fe symptom elicited by all three lines was the development of a leaf chlorosis. All lines acidified the plant medium and had induced expression of *HcIRT1* under -Fe versus +Fe. Several other -Fe symptoms were, however, only displayed by Karkar and Douiret, but not by Thelja. Karkar and Douiret exhibited quite drastic leaf chlorosis at -Fe. This was evident from accelerated leaf chlorosis and the low chlorophyll contents at – versus +Fe after 10 days of -Fe. Leaf chlorosis is a frequently occurring stress symptom in plants since under unfavorable conditions plants tend to reduce photosystem activity by removing chlorophyll and degrading chloroplasts to avoid additional stress caused by the light. This phenomenon can be measured by PAM chlorophyll fluorescence, which was lower in Karkar and Douiret and fits to the leaf chlorosis observations. Moreover, several steps in photosynthetic pigment metabolism and chloroplast ultrastructure are dependent on Fe, which explains the leaf chlorosis in the young expanding leaves after transfer of the plants to -Fe conditions. Fe deficiency resulted in a decrease of Fe contents in Karkar and Douiret expanding leaves, and hence the low Fe status can be regarded as reason for the leaf chlorosis. It is surprising that biomass production was not affected by -Fe in our experiments. We explain this partly by the fact that with ANOVA and Tukey HSD we applied the appropriate but comparably conservative statistical test for multiple comparisons that keeps the family-wise error rate (FWER) at 0.05. Hence, an increasing number of comparisons increases the Type II error (false negative) rate and thus decreases the power for the single comparisons. Perhaps, less conservative tests such as Fisher LSD or two-sample *t*-tests would have resulted in statistically significant differences. The data indicate that there was a tendency for lower values at – versus +Fe for many parameters even in Thelja, but the differences were not significant according to ANOVA/Tukey HSD.

Zn and Cu contents were affected by -Fe in Karkar and Douiret in addition to Fe contents, but not in Thelja. The reduced Fe contents are explained as primary reaction by the low amount of Fe to which the plants were exposed. However, the reduced Zn and Cu contents must have been a secondary reaction of the plant to -Fe. Normally, it would be expected that Zn contents might increase upon -Fe, because increased IRT1 would take up Zn (Li et al., 2014). Perhaps, Hedysarum plants have different capacities to regulate metal homeostasis, and this capacity differs between Thelja, Douiret, and Karkar. Differences in the regulation of Fe reductase activity, *IRT1* gene expression and metal contents between different ecotypes were also found for *M. truncatula* (Li et al., 2014). Under Fe deficiency, plants can suffer from oxidative stress (Ranieri et al., 2001; Zaharieva and Abadia, 2003; Waters et al., 2012; Ramirez et al., 2013). In *A. thaliana*, the CuSOD (copper/zinc superoxide dismutase) genes *CSD1* and *CSD2* are induced under Fe deficiency and have been suggested to replace FeSOD’s (iron superoxide dismutases) to cope with oxidative stress under iron deficient conditions (Waters et al., 2012). It can be assumed that a similar mechanism exists in leguminous plants and elevated Cu and Zn contents could contribute to the effectiveness of this mechanism. Our observation of higher Cu and Zn levels in Thelja could be one possible explanation for the increased resistance of Thelja to iron deficiency compared to Karkar and Douiret. Hence, the different efficiencies of Cu and Zn uptake under Fe deficiency in Thelja, Karkar, and Douiret could be an important distinctive factor with respect to Fe deficiency tolerance.

The stronger -Fe leaf symptoms of Karkar and Douiret suggest that these lines should sense -Fe stress stronger than Thelja. But the two lines did not activate their root Fe mobilization in a stronger manner than Thelja. Karkar reduced more Fe in the root than did Douiret and Thelja. On the other hand, medium acidification capacities were similar between the lines. Thelja and Douiret displayed higher *HcIRT1* gene expression than did Karkar. An interesting regulatory phenomenon could be seen for Thelja *HcIRT1* gene expression, which was higher at +Fe compared to Karkar and Douiret. One possible explanation is that Thelja might take up more Fe at +Fe than Karkar and Douiret, but store this Fe in the root. Upon Fe deficiency the Fe stores could be remobilized and effectively transported to the shoots. Hence, Thelja could survive better upon -Fe conditions and maintain Fe levels. Since Thelja was collected in a region with calcareous soil condition it is tempting to speculate that the constitutive *HcIRT1* expression might contribute to adaptation. On the other hand, Karkar might profit from an inefficient Fe usage, perhaps caused by ineffective internal mobilization and transport in its natural habitat with saline-sodic soil. Some abiotic stress factors induced for example by salt stress affect Fe uptake negatively, which can be explained by the toxicity of metals under water loss and the increased risk of oxidative stress (Le et al., 2016). Several proteins relevant for Fe regulation including IRT1 are controlled at post-translational level (Brumbarova et al., 2015) and hence it would be interesting to combine in the future transcriptomic in comparison to protein studies to assess the physiological activities.

The present study focussed on Fe deficiency responses, which are physiologically distinct from responses to calcareous-alkaline medium conditions. Future studies should focus on natural calcareous and saline-calcareous soil conditions. Multiple factors will differ in such experiments, including pH, soil texture, other mineral availabilities and microbial communities. Quite possibly, the ecotypes may differ in their way to mobilize Fe under additional mineral deficiencies and salt stress. Furthermore, the internal iron homeostasis regulation and allocation upon -Fe should gain attention (Schuler et al., 2012). In this respect it is noteworthy that natural variation for Fe efficiency in crops can be manifested at the level of metabolite changes, citrate concentrations for Fe-citrate movement, oxidative stress scavenging and Fe-mobilizing riboflavin patterns (Kabir et al., 2012, 2013, 2015; Jelali et al., 2014; Ben Abdallah et al., 2017). Genome sequence variation of metal homeostasis-relevant genes might account for differences in the gene expression levels or functional SNPs in coding regions. Comparing stress and -Fe responses between young and adult stages as well as under double stress may lead to better understanding of the mechanism of -Fe regulation in this leguminous species. One possibility would be to conduct comparative RNAseq. Gene expression differences between the lines can be used to build novel hypotheses on the physiological mechanisms of tolerance, which could subsequently be validated in biochemical experiments. Our work lies the ground for experiments addressing the mechanistics using the characterized Thelja and Karkar ecotypes as an extreme pair for a detailed natural variation study.

## Author Contributions

PB, TS, and CA designed the experiments. HBA carried out the experiments. PB, HBA, and HM analyzed the data. CF-S performed the metal determination. PB wrote the manuscript. HBA and HM commented on the manuscript.

## Conflict of Interest Statement

The authors declare that the research was conducted in the absence of any commercial or financial relationships that could be construed as a potential conflict of interest.
