# Supplementary material for: Natural Variation in Physiological Responses of Tunisian Hedysarum carnosum Under Iron Deficiency
Source: Front Plant Sci. 2018 Oct 2;9:1383. doi: 10.3389/fpls.2018.01383 (PMC6176081; doi:10.3389/fpls.2018.01383)
Supplement: Supplementary file 1 [file Image_1.pdf]

A

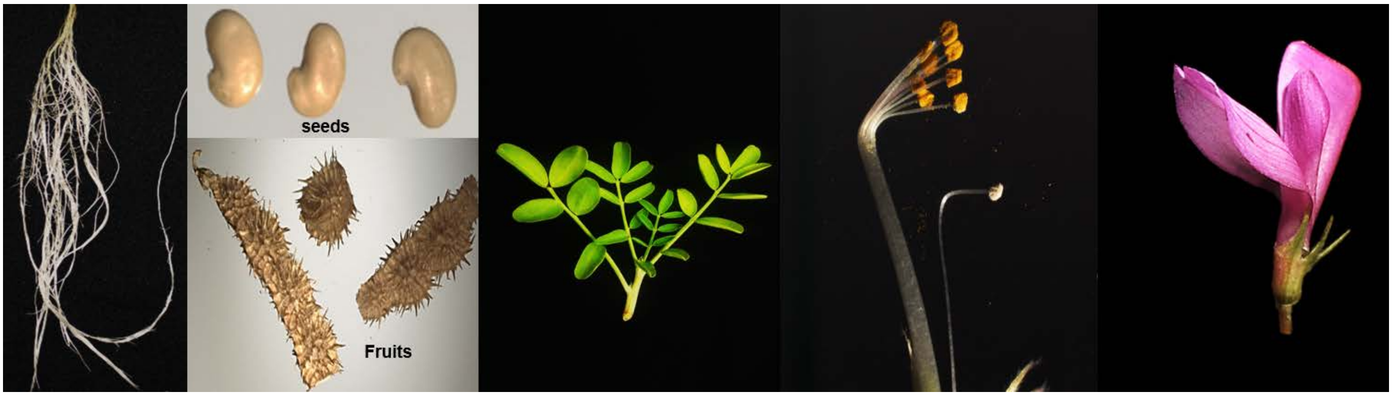

Root | Seeds and Fruits | Leaves | Papilionaceous flower

B

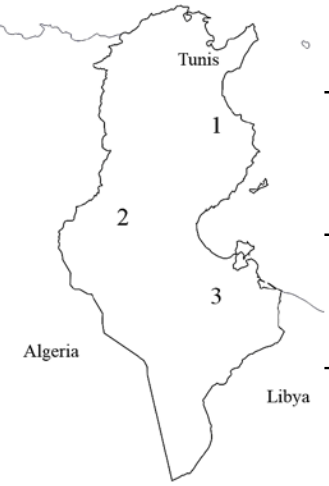

	Locality and name of line	Climate	Altitude (m)	Longitude (E)	Latitude (N)	Soil characteristics
1	Karkar	semi-arid	33	10°62'52"	35°47’34"	saline-sodic
2	Thelja	arid	195	8°19'24.2"	34°19'32.4"	calcareous
3	Douiret	saharan	434	10°17'13.1"	32°50'58.0"	sandy

Supplementary Figure 1: Overview of *Hedysarum carnosum* plant material
(A) Morphology of *H. carnosum*; (B) Geographic distribution of collected Tunisian *H. carnosum* lines.
