# Supplementary material for: Natural Variation in Physiological Responses of Tunisian Hedysarum carnosum Under Iron Deficiency
Source: Front Plant Sci. 2018 Oct 2;9:1383. doi: 10.3389/fpls.2018.01383 (PMC6176081; doi:10.3389/fpls.2018.01383)
Supplement: Supplementary file 2 [file Image_2.pdf]

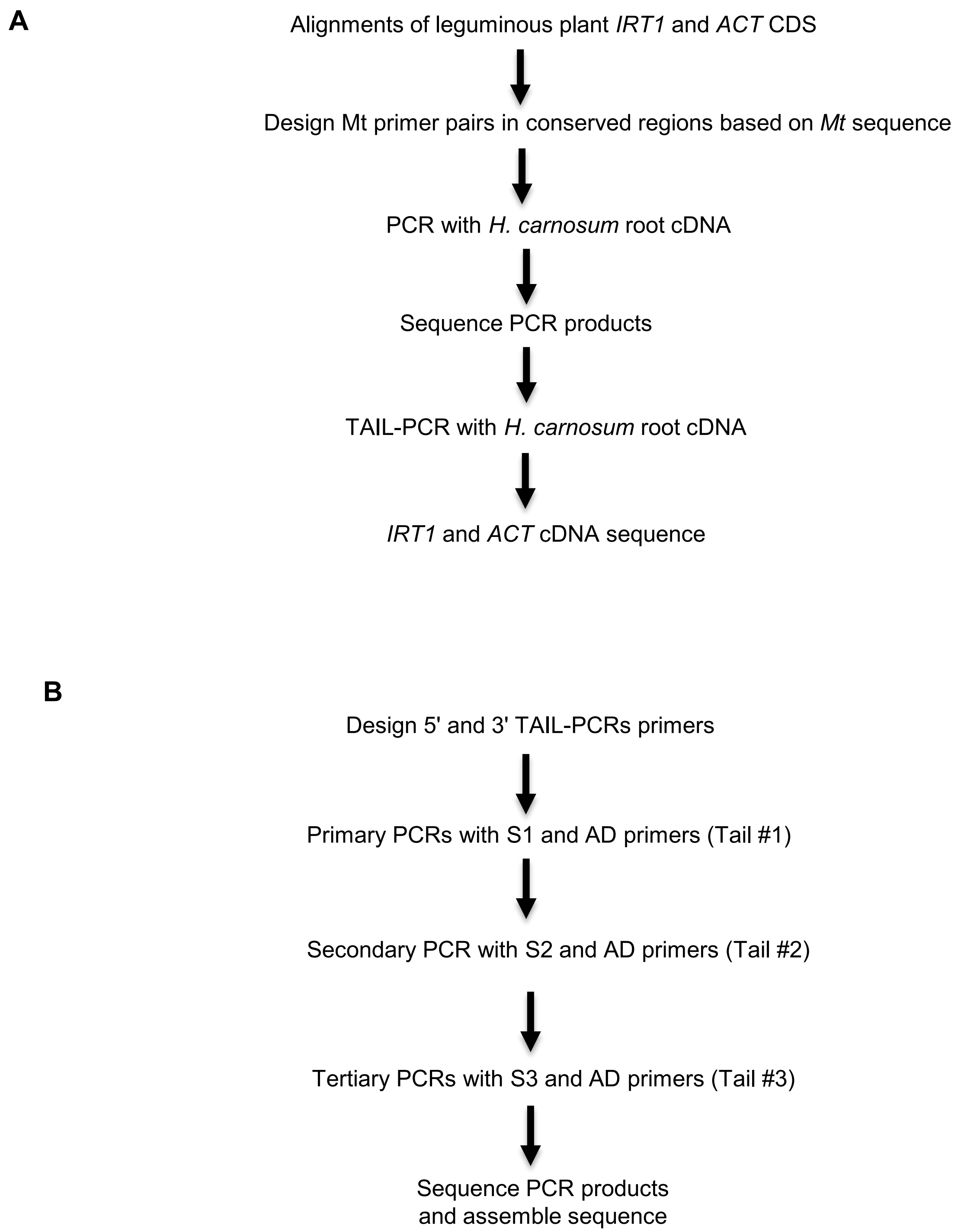

Supplementary Figure 2: Overview of *HcIRT1* and *HcACT* cDNA sequence obtention
(A) Flowchart for obtaining *H. carnosum IRT1* and *ACT* cDNA sequences; (B) Flowchart of TAIL-PCR steps.
