# Supplementary material for: Natural Variation in Physiological Responses of Tunisian Hedysarum carnosum Under Iron Deficiency
Source: Front Plant Sci. 2018 Oct 2;9:1383. doi: 10.3389/fpls.2018.01383 (PMC6176081; doi:10.3389/fpls.2018.01383)
Supplement: Supplementary file 3 [file Image_3.pdf]

Primers used in this study

1/ Mt Primers

Gene name/ Gene ID	Primer sequences (forward/reverse): (5′-3′)	T_m (°C)
MtIRT1(ZIP6)/ Mt4g083570.1	TTTAACATCACAAGCCCTAGCT<br>GCCCATTTAGCCATGAGAGA	56
MtβACT/ Mt7g093260.1	TTGCAGGAGATGATGCA<br>GTACACAGGAAATGCTTCTAA	59

2/ qRT PCR Primers

Gene name	Primer sequences (forward/reverse):(5′-3′)	T_m (°C)
HcIRT1	AGTTTCCATTTTCAGGGCTTG<br>TCTCATGTTCCCCACCTTCA	55
HcβACT	GCTACGTGTGGCTCCTGAAG<br>GGTCTCAAACATGATCTGAGTCA	55

3/ TAIL-PCR primers (gene *IRT1*)

Gene name	Primer name	Primer sequences (forward/reverse): (5′-3′)	T_m (°C)
IRT1 5′	S1	CTCCATCTTAGCAACCAGCAT	58
	S2	CGGGTTCATGCATGTCTTCC	61
	S3	TGGTCTGATTGCTTGGATGA	59
IRT1 3′	S1	TACAGCTTGGAATTGTTGTTC	59
	S2	CCCCACTTGGAATTGCCATAG	62
	S3	GAGTCCAAGGCTGCAGGTAG	60

4/ TAIL-PCR primers (gene *Beta actin*)

Gene name	Primer name	Primer sequences (forward/reverse): (5′-3′)	T_m (°C)
βACT 5′	S1	TGTGATGGTTGGTATGGGAC	59
	S2	TCTTCTAACCGAAGCACCAC	59
	S3	TGAGACCTTCAATACTCCAGC	58
βACT 3′	S1	GGAAACATTGTCTTGAGTGGTG	58
	S2	GGAGATTTCTGCATTGGCAC	59
	S3	CCACCTGAGAGGAAGTACAG	59

5/ Arbitrary degenerated Primers (AD) for TAIL-PCR

Gene name	Concentration (μM)	Primer sequences (forward/reverse): (5′-3′)	T_m (°C)
AD1	24	NGTCGASWGANAWGAA	43-48
AD2	24	TGWGNAGSANCASAGA	46-51
AD3	24	AGWGNAGWANCAWAGG	43-48
AD4	32	STTGNTASTNCTNTGC	43-51
AD5	16	NTCGASTWTSGWGTT	41-44
AD6	32	WGTGNAGWANCANAGA	40-48

Supplementary Figure 3: List of primers used in the study
